# The Role of Insects in Mechanical Transmission of Human Parasites

**DOI:** 10.5812/kowsar.20741804.2253

**Published:** 2011-09-15

**Authors:** G T El-Sherbini

**Affiliations:** 1 Department of Parasitology, Faculty of Pharmacy, October 6 University, Cairo, Egypt

**Keywords:** Fly, Cockroaches, Filth flies, Mechanical transmission

Dear Editor,

Arthropods are probably the most successful of all animals. They are found in every type of habitat and in all regions of the world. Without the vector, the parasite life cycle would be broken and the pathogen would die. By understanding how a parasite is transmitted and the involvement of vectors in the transmission, public health personnel can better design and manage control program for particular problem.

Just because an arthropod feeds on a diseased host does not ensure that it can become infected, nor does it ensure that ingested pathogens can survive and develop. [1] In mechanical transmission, the insects transport organisms on body parts, and setae that collect contamination as insect feed on dead animals or excrement. In biological transmission, there is either multiplication or development of the parasite in the arthropod or both.[2]

**Fig. 1:**
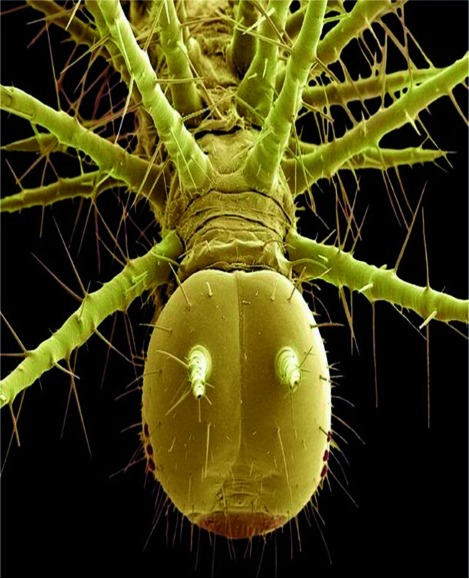
Fly after magnification of 1000,000 times by telescope (Show the small particles).

Flies have been shown to act as vectors for a number of pathogens (Figure 1). Transmission of pathogens by adult flies occurs by (i) Mechanical dislodgement from their exoskeleton that is used for adherence to vertical surfaces, (ii) Fecal deposition, and (iii) The regurgitation of incompletely digested food. Some insects infect man directly and some indirectly.

There are various mites and worms that will invade tissues. Allergies can flare from bites of bees, body lice, and bites of chiggers and ticks. Some flies will be called mechanical carries as they pick up germs by biting a diseased animal and then bite a healthy person thus contaminating them with disease.[3] Fleas carry disease after ingest plague organisms concerned with sanitation and public health as causative agents of gastrointestinal diseases in people based on synanthropy, endophily, communicative behaviour, and strong attraction to filth and human food.[4]

Cockroaches are among the most notorious pests of premises, which not only contaminate food by leaving droppings and bacteria that can cause food poisoning, but also they transmit bacteria, fungi and other pathogenic microorganisms in infested areas.[5] Their nocturnal and filthy habits make them ideal carriers of various pathogenic microorganisms. They are basically tropical insects, so far numerous pathogenic bacteria, including Salmonella spp., Shigella spp., and K. pneumoniae have been isolated from cockroaches. In addition, some parasites and fungi have been found in external surfaces or internal parts of body of cockroaches, and some studies have shown that exposure to cockroach antigens may play an important role in asthma-related health problems.[6]

Filth flies are potential mechanical vectors of disease- causing organisms because pathogens can be transferred from their contaminated bodies to our food, eyes, noses, mouths, and open wounds.[7] Myiasis is the invasion of tissues or organs of living humans or animals by fly larvae that may feed on the host’s living or dead tissue or on food ingested by the host. Host reactions may be asymptomatic, minor to violent or even death. Cockroaches and filth flies have been known to be transport hosts of Toxoplasma gondii. Insect such as dung beetles, are one of the most important food of Norway- and roof –rats and also of field mice,[8] but the role of dung beetles (Onthophagus spp.) as the carrier of coccidian oocysts is not known.

Finding and eliminating breeding places is an important first step in its control. The control or eradication of houseflies should be attempted to stop intestinal- parasite transmission in the community, in addition to drug administration. Cockroaches constitute an important reservoir for infectious pathogens; therefore, control of cockroaches will substantially minimize the spread of infectious diseases in our environment. Integrated pest control programs should involve control measures for a variety of pest species including flies, cockroaches, fleas, bedbugs, ants, and rodents. Early identification of their presence is important to avoid large infestations.
